# Comparative bioactivity analysis of latex and water extract of *Euphorbia gaillardotii*: chemical profile, antioxidant capacity, effects on water quality, *in vitro* antimicrobial‐cytotoxic activities, and *in silico* molecular docking studies

**DOI:** 10.1111/pbi.14525

**Published:** 2025-01-07

**Authors:** Mehmet Tahir Husunet, Rumeysa Mese, Esra Sunduz Yigittekin, Hasan Basri Ila

**Affiliations:** ^1^ Department of Medical Genetics, Division of Internal Medical Sciences, Faculty of Medicine Gaziantep Islamic Science and Technology University Gaziantep Turkey; ^2^ Department of General Biology, Institute of Natural and Applied Sciences Çukurova University Adana Turkey; ^3^ Department of Biology, Faculty of Arts and Sciences Çukurova University Adana Turkey

**Keywords:** *Euphorbia gaillardotii*, phytochemical analysis, antioxidant capacity, antimicrobial activity, cytotoxicity

## Abstract

The genus *Euphorbia*, belonging to the family Euphorbiaceae, represents a significant ethnobotanical heritage due to the diverse bioactive properties exhibited. In this study, the phytochemical composition and biological activities of latex and aerial parts of the water extract of *Euphorbia gaillardotii* were investigated. Phytochemical analyses were performed using gas chromatography‐mass spectrometry and high‐performance liquid chromatography techniques and total antioxidants, phenolics, sugars, organic acids, and aroma components were quantitatively determined. The effects of the test compounds on physicochemical parameters in aqueous media were evaluated by electrical conductivity, dissolved oxygen concentration, and pH measurements. Antimicrobial activity was evaluated on Gram‐positive and Gram‐negative bacteria and yeast strains using the disk diffusion method. The cytotoxic activity on the MCF‐7 human breast cancer cell line was measured spectrophotometrically using the Cell Counting Kit‐8 proliferation/apoptosis detection kit. The results showed that *E. gaillardotii* latex and aerial parts water extract significantly affected the physicochemical parameters of aqueous media, especially at high concentrations. The test substances displayed antimicrobial activity, with the latex‐impregnated disks demonstrating larger inhibition zones than the aerial parts extract. The results showed that both the latex and extract treatments exhibited concentration‐dependent effects on MCF‐7 cell viability (*P* < 0.001). Furthermore, *in silico* docking analyses revealed a robust binding affinity of succinic acid, the most prevalent bioactive compound in the extract, towards the B‐cell lymphoma 2 (Bcl‐2) molecule, with a binding energy of −6.16 kcal/mol. This may be associated with the observed cytotoxicity. These results suggest that *E. gaillardotii* may be a valuable source for potential pharmacological applications.

## Graphical abstract

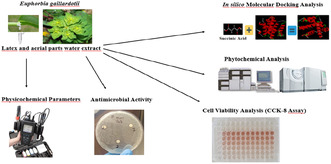

**Convention on Biodiversity:** The plant samples were procured from freely available plant products. **Botanical aspects:** Dr. Mehmet Tahir Husunet collected the specimen of *Euphorbia* from Bulanikdere Village, Silvan, Diyarbakir, Turkey. The species identification was conducted by Prof. Dr. H. Askin Akpulat through the application of macroscopic methods. The voucher sample of the plant material has been assigned the reference number AA 7580 and is deposited in the Herbarium of the Department of Biology, Faculty of Science, Sivas Cumhuriyet University.

## Introduction

The use of phytotherapeutic agents has undergone a notable evolution throughout history. Initially, whole plants or specific plant organs (such as roots, leaves, flowers, and seeds) were the primary focus of therapeutic applications. However, over time, more sophisticated formulations, including extracts, tinctures, distillates, and derivatives, have emerged as the dominant approach. At the beginning of the 21st century, 11% of the 252 pharmaceutical agents classified as essential by the World Health Organization (WHO) were reported to be exclusively angiosperm‐derived phytochemicals. Notable examples of phytochemicals with pharmacological significance include codeine, quinine, and morphine. Although the use of plant preparations as bioactive components dates back to ancient times, the integration of isolated and characterized phytochemicals into modern drug discovery and development processes is a relatively recent phenomenon, commencing in the 19th century. Natural products occupy a central position in modern pharmacology, particularly in the context of antimicrobial and antineoplastic agents. Although synthetic analogs offer advantages in terms of production cost, time efficiency, quality control procedures, and pharmacodynamic properties, there are persistent questions about their safety profile and efficacy, which has led to continued interest in phytotherapeutics (Veeresham, 2012). From the 1940s to the present, it has been reported that 48.6% of the small molecules used in oncological treatments are natural products or their semisynthetic derivatives (Newman and Cragg, 2012). In light of the historical pharmacological use of plant products, it is predicted that there will be an exponential increase in the use of these agents as a source of drugs in the future. In parallel with this, new research methodologies and therapeutic applications investigating the pharmacological effects of phytochemicals are expected to emerge.

The family Euphorbiaceae represents the fifth largest group of angiosperms and constitutes a significant contributor to the global flora, with an estimated 7500 species distributed across 300 genera (Gillespie and Armbruster, 1997). This taxon plays an important role in both traditional and modern medical practices, especially in the field of traditional Chinese medicine. The distinctive inflorescence structure and latex production of members of the Euphorbiaceae family are well‐characterized features (Salehi *et al*., 2019). The genus *Euphorbia*, classified within the Spermatophyta division, is a taxon with a global distribution. Members of this genus exhibit a wide array of morphological forms, including annual, perennial, frutescent, succulent, and arboreal. Their noteworthy ecological adaptations enable them to flourish in a vast range of habitats, encompassing sea level to an altitude of 4000 meters. The *Euphorbia* genus is well‐suited to a diverse range of ecosystems, including coastal sand formations, steep inclines, cultivated regions, saline‐rich terrain, gypsum‐laden soils, wind‐sculpted dunes, arid gravel plains, waterside zones, forest plantations, disturbed urban spaces, abandoned farmlands, and overgrazed deteriorated landscapes. This plant group is distinguished by its distinctive floral structure and unique fruit and seed attributes, which set it apart from other botanical taxa (Kaya *et al*., 2013).

In ethnomedical practices, the latex of various *Euphorbia* species has been traditionally utilized topically to address a range of dermatological and rheumatic conditions. These treatments encompass the removal of verruca vulgaris, the healing of eczematous lesions, and the provision of analgesic relief (Baytop, 1999). Detailed phytochemical investigations into *Euphorbia* species have identified several bioactive compounds, including jatrophane diterpene, jatrophane polyester (Hohmann *et al*., 2003), tigliane diterpene, cycloartane‐type triterpene (Haba *et al*., 2007), and quercetin (Chaabi *et al*., 2007). The antiviral (Betancur‐Galvis *et al*., 2002), antitumoral (Valadares *et al*., 2006), antiproliferative (Chaabi *et al*., 2007), and cytotoxic (Aslantürk *et al*., 2021; Baloch *et al*., 2008; Betancur‐Galvis *et al*., 2002; Whelan and Ryan, 2003) properties of isolated bioactive constituents from *Euphorbia* species have been substantiated through various *in vitro* and *in vivo* experimental studies. Building upon this foundation of diverse biological activities, researchers have continued to explore the potential of specific *Euphorbia* species in targeted applications, particularly in cytotoxicity and cancer research. Recent studies on *Euphorbia grandicornis* Blanc have shown significant cytotoxicity against breast cancer cell lines (MCF‐7). It is noteworthy that the root extract demonstrated selectivity towards cancer cells over non‐cancerous controls (SI = 4.80), providing further support for the proposition that *E. grandicornis* may be a potential source of anticancer compounds (Magozwi *et al*., 2021). The genus *Euphorbia* has attracted considerable interest in the field of cancer research due to the diversity of its metabolic profile and the potential for its cytotoxic properties. Recent studies have demonstrated remarkable antiproliferative effects of various *Euphorbia* species *(E. officinarum* and *E. lactea*) against several human cancer cell lines (human colon adenocarcinoma, human hepatoma, human breast adenocarcinoma), with some species exhibiting potent activity at low micromolar concentrations (El‐Hawary *et al*., 2020). In addition to its cytotoxic properties, *Euphorbia helioscopia* has demonstrated significant antimicrobial activity against a range of human pathogens. It is noteworthy that aqueous and ethanolic extracts of *E. helioscopia* exhibited potent inhibitory effects against various bacterial strains, including *Escherichia coli*, *Bacillus subtilis*, *Staphylococcus aureus*, and *Klebsiella pneumoniae*, as well as fungal species, such as *Trichoderma harzianum*, *Rhizopus nigricans*, and *Aspergillus niger*. This highlights the potential of *E. helioscopia* as a source of novel antimicrobial compounds (Waheed *et al*., 2020).

The predominant fatty acid identified in the petroleum ether extract of *E. gaillardotii* was palmitic acid. In the essential oil fraction of the same species, arachidic acid emerged as the most abundant component. The chromatographic analysis detected the presence of 27 distinct compounds, with hesperidin, rutin, hyperoside, quinic acid, malic acid, gallic acid, and tannic acid found in the highest concentrations. The methanol extract of *E. gaillardotii* exhibited significant antioxidant activity across various *in vitro* assays (Ertas *et al*., 2015).

An examination of the existing literature highlights that species within the *Euphorbia* genus display a broad spectrum of pharmacological activities. This study aimed to explore the biological activities of the latex and total extract from *E. gaillardotii*. A key aspect of our research is the evaluation of the comparative cytotoxic and cytostatic effects on the MCF‐7 human breast cancer cell line. Additionally, we investigated the influence of *Euphorbia* derivatives on physicochemical parameters, such as electrical conductivity, oxygen‐binding capacity, and pH parameters. This multidisciplinary methodology provides new insights into potential pharmaceutical applications. In conclusion, this research evaluates the therapeutic potential of *Euphorbia* species and lays the groundwork for future drug development studies. The outcomes have substantial implications for designing novel pharmaceutical concepts, developing advanced therapeutic strategies, and enriching the current scientific literature.

## Results

### Biochemical parameters and antioxidant activity findings

Our research findings revealed significant differences in the chemical composition and antioxidant activities between the latex and aerial water extracts of *E. gaillardotii*.

One of the test substances, latex, was found to have a significantly high concentration of terpenes, accounting for 99.22% of its composition. In contrast, the terpene content in the water extract was markedly low, at 1.17%. The water extract exhibited a more complex chemical profile, including alcohols (57.75%), esters (2.64%), aldehydes (0.97%), and acids (25.83%). Furthermore, the water extract was found to contain glucose (1.09%) and fructose (0.3%), however, these sugars were not present in the latex. The water extract had a high concentration of organic acids, including succinic acid (SA) (1345.99 mg/100 g), citric acid (88.92 mg/100 g), and L‐ascorbic acid (51.55 mg/100 g). The antioxidant effect was assessed using the 2,2‐diphenyl‐1‐picrylhydrazyl (DPPH) radical scavenging activity as an indicator. The water extract exhibited significantly higher antioxidant activity (63.41 ± 0.96%) compared with the latex (5.58 ± 0.69%). Although both test substances contained a high total phenol content, the water extract demonstrated a higher and statistically significant difference (724.52 ± 2.72 mg GAE/100 g) compared with the latex (601.31 ± 0.94 mg GAE/100 g) (Table 1). The findings of this study indicate that the aqueous extract derived from the aerial parts of the plant exhibits a more complex chemical composition and demonstrates enhanced antioxidant effects. It is noteworthy that the increased organic acid content and significant DPPH radical neutralizing capacity indicate that the water extracted from aerial parts may have considerable potential for antioxidant applications. The latex contained a remarkably high terpene concentration, amounting to 99.22%. The increased terpene levels detected in the latex specimen suggest its potential value as a source for antimicrobial or anti‐inflammatory applications. The identification of monosaccharides (specifically glucose and fructose) and substantial alcohol content within the aqueous extract implies a possible role in metabolic pathways. These observations indicate that different components of the *E. gaillardotii* plant contain a diverse array of bioactive substances, potentially rendering them a significant resource for future pharmacological investigations.

**Table 1:** The biochemical constituents and antioxidant properties of the latex and aerial part water extract of *E. gaillardotii*

Parameters	Latex	Extract
Total Alcohols (%)	0.17	57.75
Total Esters (%)	Not measured/not detected	2.64
Total Aldehydes (%)	Not measured/not detected	0.97
Total Terpens (%)	99.22	1.17
Total Acid (%)	Not measured/not detected	25.83
Glucose (%)	Not measured/not detected	1.09
Fructose (%)	Not measured/not detected	0.3
L‐Ascorbic acid (mg/100 g)	Not measured/not detected	51.55
Oxalic acid (mg/100 g)	Not measured/not detected	6.19
Citric acid (mg/100 g)	Not measured/not detected	88.92
Succinic acid (mg/100 g)	Not measured/not detected	1345.99
Fumaric acid (mg/100 g)	Not measured/not detected	8.01
DPPH Radical Scavenging (%)*	5.58 ± 0.69	63.41 ± 0.96 a_3_
Total phenol (mg/GAE^†^ 100 g)	601.31 ± 0.94	724.52 ± 2.72 a_3_

* The DPPH radical scavenging activity was calculated using the formula: $\frac{A\_\left\{\text{control}\right\}-A\_\left\{\text{sample}\right\}}{A\_\left\{\text{control}\right\}}\times 100$. In this context, (*A*_{control}) represents the absorbance of the DPPH solution after 30 min, whilst (*A*_{sample}) represents the absorbance after the sample has been added and incubated for 30 min.

^†^ GAE: Gallic Acid Equivalent. a_3_: The difference between the latex and the extract is statistically significant (*P* ≤ 0.001).

### Effect of test materials on water quality indicators

Latex and aerial parts extract of *Euphorbia* exhibited significant effects on water's electrical conductivity, dissolved oxygen (DO) content, and pH levels. Notably, the highest concentration applications of both latex and plant extract led to more substantial increases in conductivity. DO levels typically showed a decreasing trend in all treatments, with more marked reductions observed at higher concentrations of both latex and extract. pH values exhibited minimal fluctuations, although discernible differences emerged between distilled water, latex, and extracts. As the concentration increased, there was a corresponding increase in conductivity and a decrease in pH and DO. In general, time‐dependent changes were observed in all samples. Conductivity and pH tended to increase with increasing application time of the test substance, whereas DO tended to decrease (Table 2).

**Table 2:** Concentration‐ and time‐dependent effects of test substances (latex and extract) on conductivity, dissolved oxygen, and pH parameters

Treatment	Time (min)	Conductivity (μS/cm)	Dissolved Oxygen (mg/L)	pH
Distilled water	0	1.50 ± 0.00	5.10 ± 0.17	6.71 ± 0.13
	15	3.45 ± 0.13 b_3_	5.36 ± 0.14	6.65 ± 0.02
	30	7.92 ± 0.19 b_3_	4.77 ± 0.31	6.46 ± 0.01 b_1_
	60	12.13 ± 0.02 b_3_	5.44 ± 0.20	6.44 ± 0.02 b_1_
	120	18.22 ± 0.04 b_3_	5.36 ± 0.25	6.74 ± 0.13
	240	21.70 ± 0.03 b_3_	5.03 ± 0.13	6.88 ± 0.01
	1440	24.89 ± 0.01 b_3_	4.95 ± 0.18	7.52 ± 0.07 b_3_
0.01% Latex	0	18.89 ± 0.03 a_3_	4.87 ± 0.17	6.41 ± 0.00 a_3_
	15	109.97 ± 0.03 a_3_b_3_	5.21 ± 0.22	6.38 ± 0.00 a_3_b_3_
	30	161.90 ± 0.30 a_3_b_3_	4.91 ± 0.04	6.50 ± 0.02 a_1_b_3_
	60	307.67 ± 1.20 a_3_b_3_	4.64 ± 0.15 a_3_	6.33 ± 0.00 a_3_b_3_
	120	376.33 ± 0.33 a_3_b_3_	4.68 ± 0.04 a_2_	6.22 ± 0.00 a_3_b_3_
	240	385.00 ± 0.00 a_3_b_3_	4.96 ± 0.15	6.21 ± 0.00 a_3_b_3_
	1440	390.33 ± 0.33 a_3_b_3_	4.27 ± 0.00 a_3_b_2_	6.53 ± 0.00 a_3_b_3_
0.1% Latex	0	147.27 ± 0.06 a_3_	5.12 ± 0.16	5.97 ± 0.02 a_3_
	15	259.77 ± 0.56 a_3_b_3_	4.58 ± 0.04 a_3_b_3_	5.96 ± 0.00 a_3_
	30	333.00 ± 0.00 a_3_b_3_	4.86 ± 0.02	5.90 ± 0.00 a_3_b_3_
	60	439.00 ± 0.00 a_3_b_3_	4.62 ± 0.12 a_3_b_3_	5.93 ± 0.00 a_3_b_1_
	120	507.00 ± 0.00 a_3_b_3_	4.05 ± 0.06 a_3_b_3_	5.72 ± 0.00 a_3_b_3_
	240	513.67 ± 0.33 a_3_b_3_	4.90 ± 0.05	5.88 ± 0.00 a_3_b_3_
	1440	517.33 ± 0.33 a_3_b_3_	4.37 ± 0.08 a_2_b_3_	6.19 ± 0.00 a_3_b_3_
1% Latex	0	1019.00 ± 0.00 a_3_	4.94 ± 0.01	5.62 ± 0.02 a_3_
	15	1090.33 ± 0.33 a_3_b_3_	4.81 ± 0.00 a_2_	5.56 ± 0.00 a_3_b_3_
	30	1258.67 ± 0.66 a_3_b_3_	4.81 ± 0.17	5.58 ± 0.00 a_3_b_1_
	60	1315.33 ± 0.33 a_3_b_3_	5.07 ± 0.15	5.59 ± 0.00 a_3_
	120	1409.33 ± 1.15 a_3_b_3_	4.00 ± 0.12 a_3_b_3_	5.48 ± 0.00 a_3_b_3_
	240	1420.67 ± 0.88 a_3_b_3_	5.40 ± 0.13 b_1_	5.52 ± 0.00 a_3_b_3_
	1440	1430.00 ± 0.57 a_3_b_3_	1.63 ± 0.05 a_3_b_3_	5.72 ± 0.00 a_3_b_3_
0.01% Extract	0	45.97 ± 0.03 a_3_c_3_	5.03 ± 0.12	6.01 ± 0.05 a_3_c_1_
	15	56.03 ± 0.52 a_3_b_3_c_3_	5.01 ± 0.09	6.00 ± 0.01 a_3_c_3_
	30	74.17 ± 0.58 a_3_b_3_c_3_	4.79 ± 0.14	5.96 ± 0.00 a_3_c_3_
	60	89.43 ± 0.21 a_3_b_3_c_3_	4.68 ± 0.16 a_2_	5.94 ± 0.01 a_3_b_1_c_3_
	120	94.67 ± 0.12 a_3_b_3_c_3_	4.69 ± 0.10 a_2_	6.01 ± 0.01 a_3_c_3_
	240	99.80 ± 0.15 a_3_b_3_c_3_	4.88 ± 0.20	6.07 ± 0.01 a_3_c_3_
	1440	106.93 ± 0.03 a_3_b_3_c_3_	1.81 ± 0.04 a_3_b_3_c_3_	6.40 ± 0.01 a_3_b_3_c_2_
0.1% Extract	0	377.67 ± 0.33 a_3_c_3_	4.94 ± 0.15	5.65 ± 0.00 a_3_c_3_
	15	388.67 ± 0.33 a_3_b_3_c_3_	4.50 ± 0.13 a_3_b_1_	5.60 ± 0.00 a_3_b_3_c_3_
	30	406.67 ± 0.88 a_3_b_3_c_3_	5.06 ± 0.21	5.64 ± 0.00 a_3_c_3_
	60	411.67 ± 0.33 a_3_b_3_c_3_	4.12 ± 0.11 a_3_b_3_c_1_	5.64 ± 0.00 a_3_c_3_
	120	416.67 ± 0.57 a_3_b_3_c_3_	4.08 ± 0.10 a_3_b_3_	5.63 ± 0.00 a_3_b_3_c_3_
	240	420.00 ± 0.00 a_3_b_3_c_3_	4.82 ± 0.06	5.72 ± 0.00 a_3_b_3_c_3_
	1440	471.67 ± 0.33 a_3_b_3_c_3_	0.08 ± 0.03 a_3_b_3_c_3_	6.00 ± 0.00 a_3_b_3_c_3_
1% Extract	0	2786.67 ± 1.45 a_3_c_3_	3.95 ± 0.13 a_3_c_2_	5.33 ± 0.00 a_3_c_2_
	15	2788.67 ± 0.88 a_3_c_3_	3.51 ± 0.05 a_3_c_3_	5.33 ± 0.00 a_3_c_3_
	30	2808.33 ± 3.17 a_3_b_3_c_3_	3.78 ± 0.21 a_3_c_1_	5.33 ± 0.00 a_3_c_3_
	60	2808.67 ± 0.88 a_3_b_3_c_3_	3.44 ± 0.10 a_3_b_1_c_3_	5.33 ± 0.00 a_3_c_3_
	120	2815.33 ± 2.40 a_3_b_3_c_3_	3.46 ± 0.26 a_3_b_1_	5.35 ± 0.00 a_3_b_2_c_3_
	240	2821.67 ± 1.85 a_3_b_3_c_3_	3.02 ± 0.10 a_3_b_3_c_3_	5.31 ± 0.00 a_3_b_3_c_3_
	1440	2933.33 ± 1.33 a_3_b_3_c_3_	0.81 ± 0.15 a_3_b_3_c_3_	5.27 ± 0.00 a_3_b_3_c_3_

a: Statistically significant difference from the distilled water control.

b: Statistically significant difference from the group baseline (0 min).

c: Statistically significant difference between the latex and extract (same concentration and time point).

a_1_b_1_c_1_ ≤ 0.05, a_2_b_2_c_2_ ≤ 0.01, a_3_b_3_c_3_ ≤ 0.001.

### Antimicrobial activity of *Euphorbia gaillardotii*

The antimicrobial activity of *Euphorbia* latex and aerial parts extract was evaluated using the disk diffusion method. The findings indicated that both materials demonstrated notable antimicrobial activity. The latex‐impregnated disks exhibited a higher degree of antimicrobial activity than the water extract. The antimicrobial activity demonstrated an increase with concentration; however, this increase did not exhibit a linear relationship. The antifungal activity of the latex and extract was observed to be greater than that of nystatin, which was used as a positive control. The most susceptible microorganisms to latex were *Staphylococcus epidermidis, Pseudomonas aeruginosa, Yersinia* sp., and *Candida albicans*. The highest concentration of the water extract was effective against *Staphylococcus aureus*, *S. epidermidis*, *P. aeruginosa*, *Yersinia* sp., and *C. albicans. Escherichia coli* was resistant to both the latex and the extract. MRSA and VRE showed resistance to all treatments except the highest latex concentration (Table 3). These findings emphasize the potential antimicrobial properties of *Euphorbia* species and offer promising results for future therapeutic applications.

**Table 3:** Antimicrobial effects of the test substances (latex and extract) on Gram (+/−) bacteria and yeast, as determined by the disc diffusion method

Cons. (mg/mL)	Inhibition zone diameters by organism* (mm)										
	*E.coli*	MRSA	VRE	*S. aureus*	*S. epidermidis*	*P. aeruginosa*	*B. subtilis*	*Klebsiella* sp.	*Yersinia* sp.	*C. jejuni*	*C. albicans*
Ampicillin	6.00 ± 0.00	6.00 ± 0.00	6.00 ± 0.00	6.00 ± 0.00	14.00 ± 0.00	6.00 ± 0.00	6.00 ± 0.00	12.00 ± 0.00	6.00 ± 0.00	14.00 ± 0.00	–
Nistatine	–	–	–	–	–	–	–	–	–	–	6.00 ± 0.00
Latex											
0	6.00 ± 0.00	6.00 ± 0.00	6.00 ± 0.00	6.00 ± 0.00	6.00 ± 0.00 a_3_	6.00 ± 0.00	6.00 ± 0.00	6.00 ± 0.00 a_3_	6.00 ± 0.00	6.00 ± 0.00 a_3_	6.00 ± 0.00
0.015625	6.00 ± 0.00	6.00 ± 0.00	6.00 ± 0.00	6.00 ± 0.00	16.00 ± 0.00 b_3_	16.00 ± 0.00 a_3_b_3_	15.00 ± 0.57 a_3_b_3_	9.33 ± 3.33 b_1_	9.33 ± 3.33	11.33 ± 2.66 a_1_b_3_	16.00 ± 1.00 a_3_b_3_
0.03125	6.00 ± 0.00	6.00 ± 0.00	6.00 ± 0.00	15.33 ± 0.33 a_3_b_3_	16.00 ± 0.00 b_3_	16.33 ± 0.33 a_3_b_3_	6.00 ± 0.00	6.00 ± 0.00 a_3_	15.67 ± 0.33 a_3_b_3_	6.00 ± 0.00 a_3_	11.67 ± 2.96 a_2_b_2_
0.0625	6.00 ± 0.00	14.33 ± 0.33 a_3_b_3_	15.33 ± 0.33 a_3_b_3_	6.00 ± 0.00	16.00 ± 0.00 b_3_	6.00 ± 0.00	6.00 ± 0.00	6.00 ± 0.00 a_3_	15.00 ± 0.00 a_3_b_3_	6.00 ± 0.00 a_3_	6.00 ± 0.00
Extract											
0	6.00 ± 0.00	6.00 ± 0.00	6.00 ± 0.00	6.00 ± 0.00	6.00 ± 0.00 a_3_	6.00 ± 0.00	6.00 ± 0.00	6.00 ± 0.00 a_3_	6.00 ± 0.00	6.00 ± 0.00 a_3_	6.00 ± 0.00
0.015625	6.00 ± 0.00	6.00 ± 0.00	6.00 ± 0.00	6.00 ± 0.00	6.00 ± 0.00 a_3_c_3_	6.00 ± 0.00 c_3_	6.00 ± 0.00 c_3_	6.00 ± 0.00 a_3_c_1_	6.00 ± 0.00	6.00 ± 0.00 a_3_c_3_	6.00 ± 0.00 c_3_
0.03125	6.00 ± 0.00	6.00 ± 0.00	6.00 ± 0.00	6.00 ± 0.00 c_3_	6.00 ± 0.00 a_3_c_3_	6.00 ± 0.00 c_3_	6.00 ± 0.00	6.00 ± 0.00 a_3_	6.00 ± 0.00 c_3_	6.00 ± 0.00 a_3_	6.00 ± 0.00 c_2_
0.0625	6.00 ± 0.00	6.00 ± 0.00 c_3_	6.00 ± 0.00 c_3_	13.33 ± 0.33 a_3_b_3_c_3_	15.67 ± 0.33 b_3_	13.33 ± 3.71 a_3_b_3_c_3_	6.00 ± 0.00	6.00 ± 0.00 a_3_	11.00 ± 2.51 a_1_b_1_c_1_	6.00 ± 0.00 a_3_	15.67 ± 0.88 a_3_b_3_c_3_

* For organisms with no inhibition zone around the disc, the zone diameter is given as the disc diameter, which is 6 mm. MRSA: Methicillin‐resistant *Staphylococcus aureus*. VRE: vancomycin‐resistant Enterococcus. ‘–’: No action taken. a: The difference is significant compared with the positive control, b: The difference is significant compared with the 0 mg/mL, c: The difference is significant compared with latex of the same concentration. a_1_b_1_c_1_ ≤ 0.05, a_2_b_2_c_2_ ≤ 0.01, a_3_b_3_c_3_ ≤ 0.001.

### Cell viability findings (cytostatic/proliferative effects)

**Figure 1:**
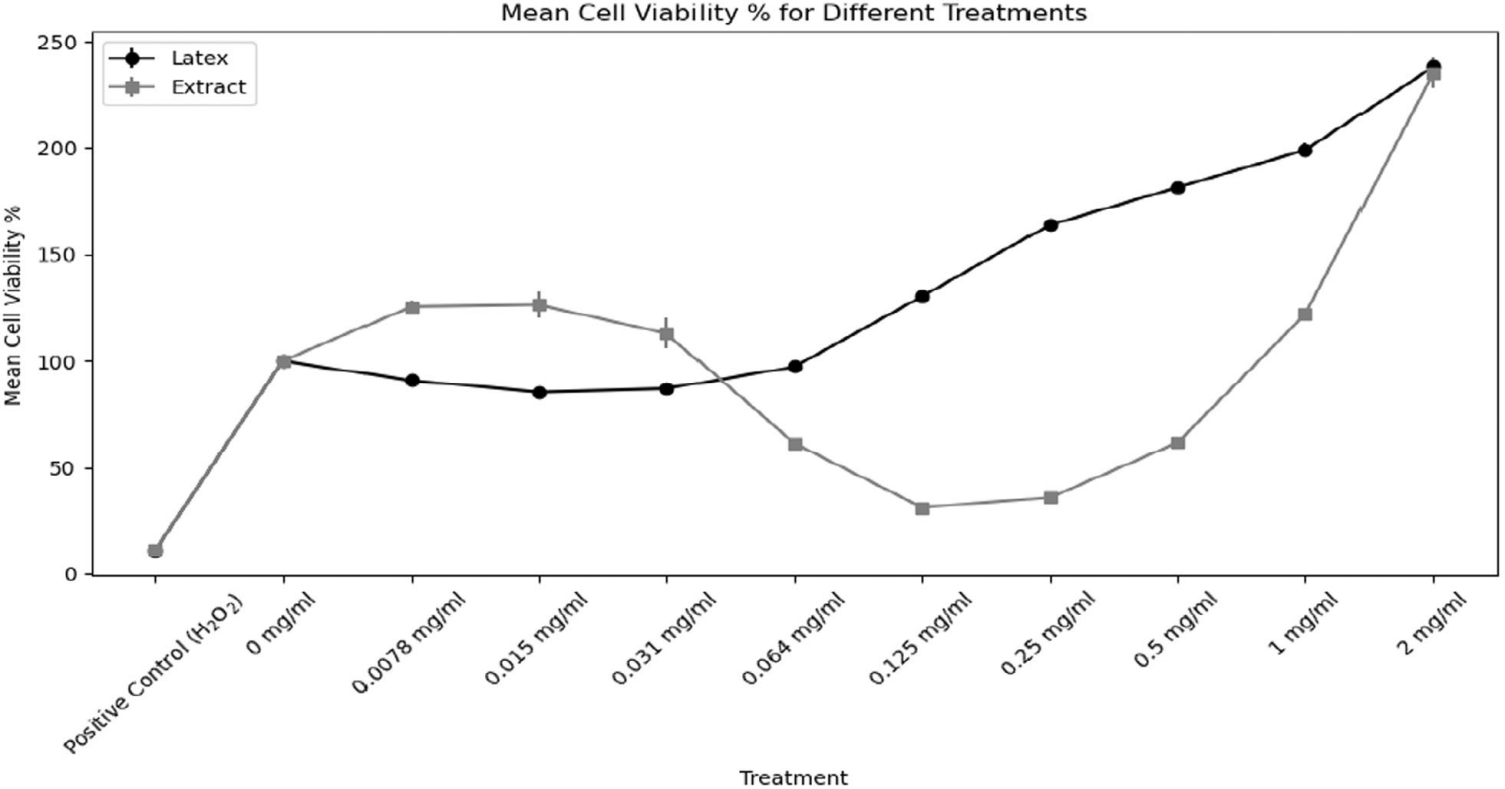
Mean cell viability values (latex and extract). This graph illustrates the mean cell viability values for different treatments with Euphorbia latex and aerial parts water extract.

The impact of *Euphorbia* latex and extract on cellular viability was investigated through the utilization of the Cell Counting Kit‐8 (CCK‐8) assay. The results revealed that the impact of both substances varied based on their concentration levels. In the latex treatment group, a notable decrease in cellular viability was observed at lower concentrations ranging from 0.0078 to 0.031 mg/mL, with statistical significance (*P* < 0.01). Nevertheless, at concentrations exceeding 0.064 mg/mL, cell viability approached the control level (*P* = 0.37). At higher concentrations (0.125–2 mg/mL), a significant increase in cell viability was observed (*P* < 0.001). The greatest increase in cell viability was recorded at a concentration of 2 mg/mL, with a 238.14% increase compared with the control group. In the extract treatment, a 125%–126% increase in cell viability was observed at low concentrations (0.0078–0.015 mg/mL) (*P* < 0.001). A decline in cell viability was observed in the concentration range of 0.031–0.064 mg/mL, reaching its lowest point at concentrations of 0.125 and 0.25 mg/mL (*P* < 0.001). At a concentration of 0.5 mg/mL, cell viability increased once more, reaching its highest point at 2 mg/mL (235.41%, *P* < 0.001). The positive control, H_2_O_2_, demonstrated a significant reduction in cell viability across both treatments. These findings substantiate the dependability of the experimental system. The statistical analysis revealed that there were substantial differences in cell viability between the latex and extract treatments (*P* < 0.001) (Table 4; Figure 1). These findings illustrate the intricate impact of *Euphorbia* latex and water extract on cellular viability, underscoring the necessity for further investigation into their potential therapeutic applications.

**Table 4:** Comparison of viability rates of the MCF‐7 cell line treated with latex and extract for 24 h, as determined by CCK‐8 test results

Treatment	Mean cell viability (%)	
	Latex	Extract
Positive Control (H_2_O_2_)	11.228 ± 0.39	11.340 ± 0.66
0 mg/mL	100.000 ± 0.00 a_3_	100.000 ± 0.00 a_3_
0.0078 mg/mL	90.890 ± 2.21 a_3_b_2_	125.453 ± 3.16 a_3_b_3_
0.015 mg/mL	85.533 ± 2.52 a_3_b_3_	126.338 ± 6.06 a_3_b_3_
0.031 mg/mL	87.093 ± 2.14 a_3_b_3_	112.998 ± 7.26 a_3_b_1_
0.0625 mg/mL	97.288 ± 0.57 a_3_	61.368 ± 1.46 a_3_b_3_
0.125 mg/mL	130.248 ± 1.41 a_3_b_3_	31.300 ± 2.48 a_3_b_3_
0.25 mg/mL	163.953 ± 1.01 a_3_b_3_	35.890 ± 1.19 a_3_b_3_
0.5 mg/mL	181.585 ± 1.86 a_3_b_3_	61.750 ± 2.03 a_3_b_3_
1 mg/mL	198.900 ± 3.07 a_3_b_3_	121.905 ± 1.29 a_3_b_3_
2 mg/mL	238.143 ± 4.01 a_3_b_3_	235.410 ± 7.35 a_3_b_3_

a: Significant difference compared with positive control (H_2_O_2_); b: Significant difference compared with 0 mg/mL.

a_1_b_1_ ≤ 0.05, a_2_b_2_ ≤ 0.01, a_3_b_3_ ≤ 0.001.

### *In silico* molecular docking data

**Figure 2:**
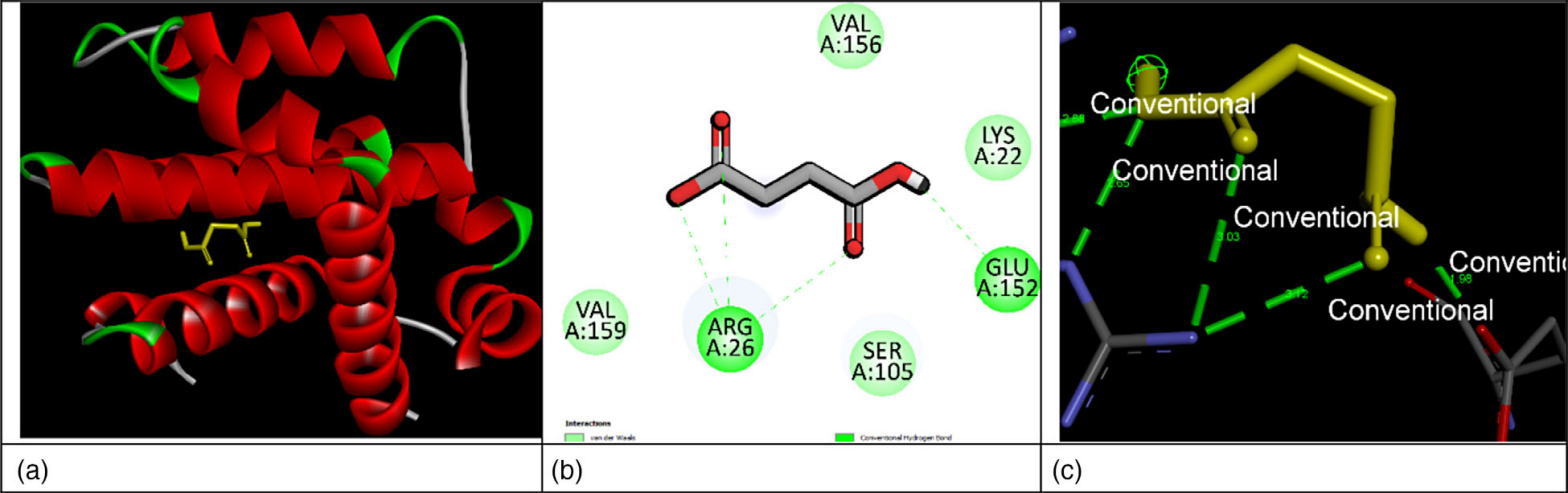
*In silico* molecular docking analysis of succinic acid binding to B‐cell lymphoma 2 (Bcl‐2) protein. (a) The optimal docking conformation of SA within the Bcl‐2 binding pocket. (b) A two‐dimensional illustration of the intermolecular interactions between SA and the amino acid residues of Bcl‐2. (c) A three‐dimensional representation of the receptor‐ligand interaction is presented, with the hydrogen bond surface depicted.

The interaction of SA with the Bcl‐2 protein was evaluated in accordance with the Gibbs free binding energy (ΔG binding). The interaction of SA with both the transpeptidase enzyme involved in bacterial wall synthesis and the Bcl‐2 protein that regulates apoptosis was analyzed. The molecular docking‐free energy analysis revealed that while the binding interaction between SA and transpeptidase remained proximal to the threshold value at −5.54 kcal/mol, the interaction with Bcl‐2 protein demonstrated a significantly enhanced binding energy, registering below the threshold at −6.16 kcal/mol. Therefore, SA exhibits a stronger docking affinity with Bcl‐2 than that observed with transpeptidase. The threshold value is −6.00 kcal/mol, as established by Shityakov and Förster (2014). The optimal docking position is illustrated in Figure 2a. In the interaction between the Bcl‐2 protein and SA, three hydrogen bonds were formed with amino acid ARG26 and one hydrogen bond with amino acid GLU152. Additionally, van der Waals bonds were observed with amino acids VAL156, VAL159, SER105, and LYS22 (Figure 2b,c).

## Discussion

This study thoroughly investigated the biological and physicochemical properties of both the latex and the aerial parts water extract of *E. gaillardotii*. The main aim was to identify the bioactive components and assess the antioxidant properties of these plant materials. The study also explored their effects on physicochemical parameters, including water's electrical conductivity, DO, and pH, as well as their antimicrobial and apoptotic/proliferative profiles. The findings provided new and significant insights not previously reported in the literature. Additionally, some results were consistent with those of previous studies on other *Euphorbia* species. This research represents a notable advancement in understanding the bioactive potential and therapeutic applications of *E. gaillardotii*.

Significant differences were observed in the chemical composition and antioxidant capacity between the latex and water extract samples from *E. gaillardotii*. The latex was found to have a high concentration of terpenes, whilst the water extract presented a more diverse chemical profile, including various organic compounds and sugars. Additionally, the water extract demonstrated greater antioxidant activity and higher phenol content. These findings indicate that the two samples may exhibit distinct biological activities and potential applications. Previous research has shown that extracts from different *Euphorbia* species can differ in chemical composition and biological effects. Although each species is unique, the variations in contents from different parts of the plant align with the results of our study (Alruhaimi *et al*., 2023; Aslantürk *et al*., 2021; Das *et al*., 2022; Saleem *et al*., 2014).

The results of our study suggest that the physicochemical characteristics of aqueous media were significantly influenced by the presence of *Euphorbia* latex and aerial parts extract. At higher concentrations, both the latex and the extract increased electrical conductivity by raising the number of dissolved ions in the solution. However, the extract had a more prominent impact on conductivity. Additionally, the experiments revealed a general decrease in DO levels, which is vital for aquatic life, especially at higher concentrations of the test substances and with prolonged exposure times. The concentration of plant extracts tested in the study had a greater effect on reducing DO levels compared with the same concentrations of latex. The observed reduction in DO levels may be due to oxygen‐consuming reactions involving components of *Euphorbia* in the aqueous solution. The difference in DO levels between the latex and the extract can be attributed to the oxygen‐binding capacity of their respective constituents. Additionally, our study observed minor fluctuations in pH levels, with the latex exhibiting slightly elevated pH values in comparison to the extract. The minimal pH changes suggest that *Euphorbia* constituents might have buffering capabilities. In a separate study employing *Moringa oleifera* seeds, a notable elevation in DO levels in aqueous media was discerned, whereas no considerable alterations in pH, conductivity, salinity, or total dissolved solids were identified (Shan *et al*., 2017). Conversely, the implementation of elevated concentrations of *Euphorbia* constituents and prolonged treatments led to a further decline in DO levels and an augmentation in conductivity, as observed in our study. In some human communities, traditional fishing methods employ the use of extracts derived from *Euphorbia* plants. The application of *Euphorbia* extract has been documented to inhibit acetylcholine esterase activity, which has been indicated as a potential cause of fish mortality in aquatic ecosystems (Benrit Vimal and Sam Manohar Das, 2015). Another potential mechanism is the reduction in DO levels in the water, which can result in hypoxic conditions for fish. Prior scientific studies have confirmed the ability of *Euphorbia* extract to bind DO (Akpa *et al*., 2009; Idowu *et al*., 2019). This evidence suggests that fish exposed to hypoxic stress may experience impaired neuromuscular coordination, increasing their susceptibility to predation.

The antimicrobial activity assays revealed that the latex exhibited marginally higher antimicrobial efficacy compared with the water extract. The antimicrobial and antioxidant activities of total extracts or isolated bioactive components of various *Euphorbia* species have been frequently highlighted in the relevant literature. The antimicrobial activity of *Euphorbia* has been observed to disrupt the permeability of the bacterial cell membrane, thereby facilitating the access of hydrophobic antibiotics to the cell. The disruption of the cell membrane resulted in the release of potassium ions and nucleotide leakage in some resistant pathogens. Similarly, it has been stated that some *Euphorbia* extracts exhibited selective antibacterial activity against gram‐positive strains but minimal activity against tested fungal strains (Ahmad *et al*., 2017; Hlila *et al*., 2017; Omar *et al*., 2024; Perumal *et al*., 2017). Furthermore, De Oliveira *et al*. (2015) found that the aqueous extract and latex preparation of *Euphorbia tirucalli* L. exhibited antifungal activity against the opportunistic yeast *Cryptococcus neoformans* strains *in vitro*.

The results of our experiments revealed that the latex and above‐ground extract treatments exhibited disparate effects on cell viability. Whilst latex displayed a proliferative effect in the estrogen‐positive receptor MCF‐7 cell line, the extract evinced a more intricate response in the same cell type. The extract was observed to exert a cytotoxic effect at moderate concentrations, whilst at both low and high concentrations, it was found to enhance cell viability. In this assay, the latex demonstrated a biphasic effect. The viability of cells treated with latex was found to decrease at low concentrations and increase at high concentrations. In contrast, the air part extract demonstrated increasing cell viability at low and high concentrations and decreasing viability at medium concentrations. This indicates a non‐monotonic dose‐response relationship. These findings deviate from the predictions of classical dose‐response models, suggesting the potential involvement of more intricate biological mechanisms. The effect shown by latex points to the opposite of the classical hormetic effect. Such opposite effects can be described in the literature as ‘reverse hormesis’ or ‘paradoxical hormesis’. The three‐step effect shown by the extract is a rarer phenomenon known as ‘triphasic dose‐response’. Such complex dose‐response relationships reflect the dynamic nature of biological systems and reveal that substances may act through different biological mechanisms at different concentrations. These observations align with the concept of hormesis, which describes U‐shaped dose responses in toxicology (Calabrese *et al*., 2007).

These findings offer valuable insights into the potential therapeutic applications of latex and extracts. However, the impact of alterations in conductivity, DO, and pH levels on cellular viability in response to test substances may be more intricate and indirect. Such alterations in these parameters may have indirectly affected viability rates by modifying the cellular environment. Nevertheless, further research, particularly *in vivo* studies, is necessary to elucidate the mechanisms underlying these effects.

The research on the cytotoxic effects of *Euphorbia* species has yielded inconclusive results. Schall *et al*. (1991) observed that Chinese hamster ovary cells exhibited no evidence of cytotoxicity when exposed to *Euphorbia splendens* latex at concentrations up to 200 μg. In contrast, De Sousa Araújo *et al*. (2015) reported that whilst the ethanolic extract of *Euphorbia hyssopifolia* L. exhibited no cytotoxicity in HepG2 cells when assessed using the MTT assay, a concentration of 1.0 mg/mL resulted in significant cellular damage as determined by the micronucleus test. Recent studies have yielded conflicting results regarding the effects of different *Euphorbia* species on cancer cells. Salah *et al*. (2001) reported that methanol extracts of the aerial parts of *Euphorbia nubica* and *Euphorbia helioscopia* contained fractions, specifically tiglian and ingenan diterpene esters, that exhibited tumour‐promoting properties and acted as non‐genotoxic carcinogens. However, Al‐Faifi *et al*. (2017) observed that the extract of *Euphorbia triaculeata* exhibited significant cytotoxicity against cancer cells. Ping *et al*. (2012), using the *Allium cepa* test, showed that *Euphorbia hirta* methanolic extract at a concentration of 1000 μg/mL decreased the mitotic index and increased chromosomal aberrations in a dose‐dependent manner, confirming its potent cytotoxic and genotoxic properties. The study, which investigated the apoptogenic potential of the methanol extract of *Euphorbia peplus* (EPME) in rats, included a series of *in vivo* heart and kidney assays in addition to *in silico* approaches. The *in silico* results indicated that the di‐(2‐ethylhexyl) phthalate component of the extract showed a higher docking affinity and subsequent inhibition at the mouse double minute 2 homologue‐p53 (MDM2‐p53) interface compared with the standard reference compound. This hypothesis was experimentally confirmed by observing strong p53 expression in kidney and heart tissues. Indeed, renal and cardiac p53 expression was significantly increased in EPME‐treated rats, whilst cardiac TNF‐α expression showed moderate levels (Abd‐Elhakim *et al*., 2019). Among the 15 diterpenoids from *Euphorbia nerifolia*, compound 6 showed a moderate cytotoxic effect in HepG2 and multidrug‐resistant HepG2/Adr cells. Compound 15 showed significant cytotoxicity against the HepG2 cell line, but was ineffective against the HepG2/Adr cell line (Li *et al*., 2019). Three novel jatrophan diterpenoids (euphoheliphanes A, B, C) isolated from *E. helioscopia* showed cytotoxic activity against six renal cell carcinoma lines (Zhou *et al*., 2021). In the study by Chen *et al*. (2021), seven compounds isolated from *E. peplus* showed no cytotoxic activity against five human tumour cell lines. Ingol‐3,7,12‐triacetate‐8‐benzoate, derived from *Euphorbia royleana*, demonstrated a modulatory effect on the HepG2/DOX cell line with P‐glycoprotein‐mediated multidrug resistance whilst exhibiting no cytotoxic properties. This effect enhanced the efficacy of doxorubicin by ~ 105 times that of verapamil (Shaker *et al*., 2020). A novel tetrahydrofuran containing two cyclomyrcinol macrocyclic diterpenes, copetdaghinane A and B, and a hemiacetal group was isolated from the above‐ground parts of *Euphorbia kopetdaghi*, a species native to the northeastern regions of Iran. The apoptotic effect of cyclomyrcinol was found to be linked to the production of reactive oxygen species (ROS) and loss of mitochondrial membrane potential (ΔΨm). In particular, copetdaghinane A was demonstrated to impede the proliferation of MCF‐7 breast cancer cells via the activation of the mitochondrial apoptotic pathway (Riahi *et al*., 2020). The chloroform fraction of the methanol extract of *Euphorbia milii* (Em‐C) showed considerable antioxidant activity. Em‐C exhibited notable cytotoxicity in comparison to 5‐fluorouracil (5‐FU) against the hepatocarcinoma (HepG2) cell line yet displayed no substantial impact on the human cervical cancer cell line (HeLa) (Chohan *et al*., 2020). GRC‐2, a prostratin analog derived from *Euphorbia grandicornis*, has been demonstrated to impede the proliferation of human non‐small cell lung cancer (NSCLC) (A549) cells through the hyperactivation of the extracellular signal‐regulated kinases (ERK) as well as the activation and nuclear translocation of protein kinase C (PKC) delta and delta‐like protein kinase (PKD). Flow cytometry analysis indicated that GRC‐2 and prostratin induced apoptosis by inhibiting the progression of the cell cycle at the G2/M phase (Tsai *et al*., 2020). The ingenol compound (IngC) derived from *E. tirucalli* demonstrated dose‐dependent cytotoxicity and colony inhibition in a panel of glioma cell lines. IngC modulated cell survival and the cell cycle by inducing S‐phase arrest through the overexpression of the p21CIP/WAF1 pathway and induced autophagy. However, it had no effect on cell migration, invasion, and apoptosis (Silva *et al*., 2019). In a study conducted by Mahmoudian‐Sani and Asadi‐Samani (2020), it was observed that the cytotoxic effect of *Euphorbia microsciadia* on the breast cancer cell line (MDA‐MB‐231) increased in a dose‐dependent manner. The underlying mechanism responsible for this cytotoxic/apoptotic effect was attributed to an elevated expression level of Let‐7, miR‐15, miR‐16, miR‐29, and miR‐34a microRNAs.

This study provides comprehensive insights into the biological and physicochemical properties of the latex and water extract from the aerial parts of *Euphorbia gaillardotii*. The results demonstrate complex dose‐response relationships, including biphasic and triphasic effects on cell viability, alongside significant alterations in the physicochemical properties of water. These findings, consistent with the hormesis concept, point to the involvement of intricate biological mechanisms. Whilst this study highlights the therapeutic potential of *E. gaillardotii*, it also emphasizes the necessity for further research to fully elucidate the underlying molecular pathways and rigorously evaluate the safety and efficacy of its potential applications. These results lay the groundwork for future investigations into the bioactive properties of this species.

## Conclusion

This study on *Euphorbia gaillardotii* reveals notable differences in chemical composition and bioactivity compared with other *Euphorbia* species. Our investigation of the latex and aerial parts extract yielded several key findings. The latex exhibited high terpene content, whilst the extract showed a more complex, heterogeneous profile. The extract demonstrated superior DPPH radical scavenging activity and higher total phenolic content compared with the latex. Additionally, the extract more significantly enhanced electrical conductivity and reduced DO content compared with the latex.

In terms of biological activity, the latex showed slightly higher antimicrobial efficacy than the water extract. Both the latex and water extract exhibited concentration‐dependent effects on cell viability, with the water extract showing a more complex pattern of influence. *In silico* molecular docking simulations with succinic acid, the most abundant molecule in the extract, suggest that the observed antimicrobial effect is unlikely due to transpeptidase inhibition. However, succinic acid's strong binding affinity for the Bcl‐2 protein in eukaryotic cells may contribute to apoptosis initiation.

Notably, our findings on the cytotoxic effects of *E. gaillardotii* extracts provide intriguing insights into their potential anticancer properties. The concentration‐dependent impact on cell viability, particularly the complex pattern observed with the water extract, suggests a selective cytotoxic effect that could be exploited in cancer therapy. Furthermore, the potential apoptosis‐inducing properties, as indicated by the *in silico* studies with succinic acid, open new perspectives for future cancer treatment strategies. These results warrant further investigation into *E. gaillardotii* as a promising source of novel anticancer compounds.

The notable reduction of the DO concentration by the extract presents a promising avenue for further research in pharmacology and industrial applications. Nevertheless, this study is predominantly based on *in vitro* and *in silico* analyses, and further *in vivo* research is essential to validate these findings in biological systems. Furthermore, the underlying molecular mechanisms that underpin the observed effects, particularly the reduction in DO and the selective cytotoxicity, necessitate further investigation. The distinctive biological activities of *E. gaillardotii* extracts indicate their potential for utilization in pharmaceuticals, particularly in the development of new anticancer therapies, as well as in agriculture and industry.

## Experimental procedures

### Plant material and extraction

**Figure 3:**
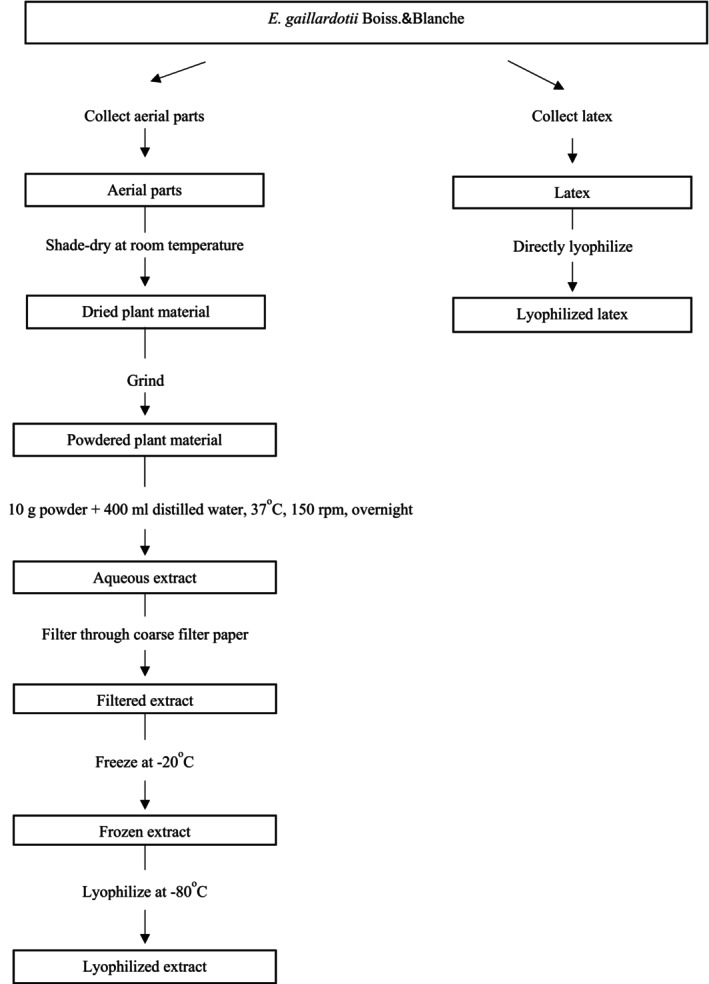
Presents a schematic representation of the extraction process for *E. gaillardotii*.

The latex and aerial parts of *E. gaillardotii* Boiss. & Blanche were collected in July from the countryside of Diyarbakir city, Turkey. The methodology for the preparation of latex and total aqueous extract of *E. gaillardotii* is detailed in Figure 3.

The aerial parts of *Euphorbia gaillardotii* were harvested, shade‐dried at room temperature, and pulverized into a fine powder. For the purpose of aqueous extraction, 10 grams of the powder were mixed with 400 milliliters of distilled water. This mixture was maintained at 37 °C and stirred at 150 revolutions per minute for 24 h. The resulting aqueous extract was filtered through coarse filter paper, frozen at −20 °C, and lyophilized at −80 °C to obtain the final lyophilized extract. Separately, the latex of the plant was collected in its natural habitat and directly lyophilized to produce lyophilized latex.

### Phytochemical analysis

The phytochemical constituents of *Euphorbia* latex and aerial extract were analyzed using two analytical systems: a gas chromatography/mass spectrometry (GC/MS) system (QP2010, Shimadzu, Kyoto, Japan) and a high‐performance liquid chromatography (HPLC) system (Prominence LC‐20A, Shimadzu, Kyoto, Japan). The phytochemical analyses for oxidative potential, total phenolic compounds, sugars, organic acids, and aroma components were conducted in the laboratories of Çukurova University, Faculty of Agriculture, Department of Horticulture.

### Physicochemical properties

The electrical conductivity, DO concentration, and pH changes in the aquatic environment were measured at 15, 30, 60, 120, 240, and 1440 min (24 h) after the test substances were added to distilled water using a Thermo Scientific Orion Star pH/ISE/conductivity/RDO/DO portable meter. The concentrations (0.01%, 0.1%, and 1%) were determined through preliminary tests. These represent the lowest doses and their multiples at which changes in electrical conductivity, DO concentration, and pH parameters were observed in aqueous media.

### Antimicrobial activity

The minimum inhibition concentration (MIC) was determined using the liquid microdilution method, as recommended by the Clinical and Laboratory Standards Institute (CLSI). Luria‐Bertani (LB) broth was employed for bacterial cultures, whilst Sabouraud dextrose broth (SDB) was utilized for yeast cultures. The bacterial strains were incubated on Mueller‐Hinton agar (MHA) plates at 37 °C for 18–24 h. Based on the results of preliminary tests, the concentrations of the test substance were prepared by serial dilution from a stock solution of 2 mg/mL to a final concentration of 0.015625 mg/mL. The incubation period for bacteria was 24 h at 37 °C, whilst that for yeasts was 48 h at 30 °C.

### Cell viability analysis

The viability of MCF‐7 cells was assessed using the Cell Counting Kit‐8 (CCK‐8) assay. The MCF‐7 cell line was cultured in 96‐well microplates at 37 °C, 95% humidity, and 5% CO_2_ for 24 h. *Euphorbia gaillardooti* latex and extract concentrations were selected based on the OECD Test No. 487 ‘*In Vitro* Mammalian Cell Micronucleus Test’ guideline (OECD, 2023). The test substances were then applied to the cells for an additional 24 h. Following the addition of the CCK‐8 reagent (Elabscience® Enhanced Cell Counting Kit 8), the cells were incubated for a period of 1–4 h, and the absorbance was then measured at 450 nm using a spectrophotometer. The cell viability rate was calculated according to the formula below:

$$\text{Cell viability rate}\;\left(\%\right)=\frac{A450\;\left(\text{sample}\right)-A450\;\left(\text{blank}\right)}{A450\;\left(\text{control}\right)-A450\;\left(\text{blank}\right)}\times 100$$

### *In silico* docking analysis

Molecular docking analyses were conducted using AutoDock 4.0 to ascertain the binding affinity and potential binding sites of SA, the most abundant biocomponent in *Euphorbia* above‐ground extract, with Bcl‐2 protein (PDB ID: 6GL8) and bacterial transpeptidase enzyme (PDB ID: 7ONO). The receptor and ligand molecule conformations were obtained from the Protein Data Bank (PDB) and prepared with AutoDockTools. The non‐polar hydrogens were combined, and Gasteiger charges were calculated. A grid box with a grid spacing of 0.375 Å and a size of 60 × 60 × 60 Å was employed. Following 25 generations of the genetic algorithm and 100 independent docking runs, the conformations exhibiting the lowest binding free energy were selected. Subsequently, the most favorable docking poses were subjected to further analysis using the BIOVIA Discovery Studio Visualizer 2016 (Husunet *et al*., 2022).

### Statistical analysis

Before analysis, the data underwent normality testing using the Shapiro–Wilk test and homogeneity of variance testing with Levene's test. The Shapiro–Wilk test results indicated a *P*‐value greater than 0.05, confirming that the data followed a normal distribution, thus eliminating the need for data normalization. A one‐way analysis of variance (ANOVA) was then performed to determine the mean differences between the various groups, followed by Fisher's least significant difference (LSD) test for post hoc pairwise comparisons. The threshold for statistical significance was set at *P* ≤ 0.05. The results are listed as mean ± standard error (SE). All statistical analyses were performed using IBM SPSS Statistics, version 25.0 (IBM Corp., Armonk, NY).

## Author contributions

**Mehmet Tahir Husunet:** Collection of plant materials, execution of extraction procedures, performance of phytochemical analyses, conduction of cell viability tests, and assessment of results. **Rumeysa Mese:** Examination of the impact of latex and extract treatments on electrical conductivity, dissolved oxygen concentration, and pH values in aqueous media and assessment of the findings. **Esra Sunduz Yigittekin:** Detection of antimicrobial activity through disk diffusion testing and assessment of the findings. **Hasan Basri Ila** was responsible for the conceptualization and design of the study, the execution of the entire calculation and statistical analysis, and the preparation and revision of the manuscript. In his capacity as the corresponding author, he was the primary coordinator and manager of the study. All authors have read and approved the final version of the manuscript.

## Funding

Sponsor along with grant number: This work was supported by the Çukurova University Scientific Research Projects (CUBAP) Coordination Unit with project code FBA‐2021‐13694.

## Conflict of interest

The authors affirm that they have no conflicts of interest regarding the completion of this work and its subsequent publication.

## Declaration of generative AI

Generative AI and AI‐enabled technologies were employed exclusively for the purpose of improving the readability and linguistic quality of the article during the writing process.

## Data Availability

The data that support the findings of this study are available on request from the corresponding author. The data are not publicly available due to privacy or ethical restrictions.
